# Outlook for modern cooking energy access in Central America

**DOI:** 10.1371/journal.pone.0197974

**Published:** 2018-06-08

**Authors:** Shonali Pachauri, Narasimha D. Rao, Colin Cameron

**Affiliations:** Energy Program, International Institute for Applied Systems Analysis (IIASA), Laxenburg, Austria; Humboldt State University, UNITED STATES

## Abstract

The Central American nations of Guatemala, Honduras and Nicaragua are among the poorest in the Americas. While the fraction of population dependent on solid fuels has declined in these nations over the last 25 years, the number of people using them has risen. Here, we first assess current patterns of cooking energy use in these nations. We then apply a discrete model of household cooking choices and demand to simulate future pathways of clean cooking uptake and the outlook for achieving target 7.1 of the Sustainable Development Goals (SDG), which aims to ensure universal access to affordable, reliable and modern energy services by 2030. We find that by 2030, ensuing income growth is likely to enable 90% of urban populations in these nations to switch to using modern cooking energy services. However, without supporting policies, between 40% to 50% of rural Guatemalans and Hondurans, while over two-thirds of rural Nicaraguans, are likely to find clean fuels or stoves unaffordable in 2030. A targeted subsidy on modern fuels, like liquid petroleum gas (LPG), is the most effective policy mechanism we studied that could provide such support. A 50% subsidy policy on LPG targeted to the rural and urban poor population could, by 2030, make cooking with LPG affordable to an additional 7.3 million people in these countries. We estimate that such a policy would cost about $250 million per year and would have negligible greenhouse gas emissions impacts. Such a policy could also have significant health benefits, preventing about 8,890 premature deaths annually from reduced exposure to cooking-related household pollution in 2030.

## 1 Introduction

The Central American nations of Guatemala, Honduras and Nicaragua are among the poorest countries in the Americas. Existing literature analyzing household cooking fuel transitions have focused largely on Asia and Africa to the neglect of nations in Central America [1]. This is despite dependence on solid fuels for cooking in rural areas of Central America similar to parts of rural Asia and Africa [2]. Recent trends suggest that between 1990 and 2015, the fraction of population dependent on solid fuels in these three countries declined from 68 percent to 57 percent. However, over the same period the total number of people dependent on solid fuels rose from 12.2 million to 13.2 million [3]. This was because population growth in the region outpaced the transition away from solid fuel cooking. For the region as a whole, traditional biomass, especially fuelwood for cooking, continues to account for more than a third of total final energy consumption, on average [4]. However, in these three countries, it accounts for a larger share of national final energy consumption, between 40‐60 percent (Table 1).This poses a challenge to achieving target 7.1 of the United Nations Sustainable Development Goals [5] that aims by 2030, to ensure universal access to affordable, reliable and modern energy services.

**Table 1:** Basic indicators for Guatemala, Honduras and Nicaragua in 2011/2012.

Indicator	Units	Guatemala	Honduras	Nicaragua
**Area**	sq. km.	107160	111890	120340
**Population**	millions	14.71	7.78	5.91
**Density**	persons/sq. km.	137	70	49
**Urbanization**	%	50	52	58
**GDP/capita (PPP)**	2011$	6957	4345	4215
**Gini Index**	Index	52	57	53
**Poverty gap at $2 a day (PPP)**	%	11	13	14
**Urban high income (>$5 a day) population**	%	40	15	36
**Electrified population**	%	82	83	78
**Population using non-solid fuels**	%	43	49	46
**Biomass share in total final energy use**	%	56	43	64
**Residential share in total final energy use**	%	60	47	70

While a vast literature exists on understanding household cooking energy patterns and choices in different regions, fewer studies have proposed methods that allow for a forward looking analysis of future scenarios. Modeling and analyses of cooking fuel choices and demand are important to understand the impacts of future cooking transitions on human wellbeing and the environment and to inform policy design [6,7]. The few studies that include forward looking scenarios have either econometrically estimated parameters influencing household cooking fuel choices [8,9], or used least-cost optimization to determine optimal cooking energy choices [10,11]. However, these approaches are limited in their ability to account for multiple cooking fuel use and fuel stacking that is a regular feature of household energy use patterns in many developing countries [12]. The model we employ allows for fuel stacking and is calibrated to data from household surveys that reflect actual cooking energy choices and patterns, shaped by preferences and behaviors in these nations.

In this work, we carry out an analysis of household cooking energy patterns in the three above mentioned Central American countries, and assess scenarios to improve access to modern cooking energy services by 2030. We contribute specifically to assessing the outlook for increasing the proportion of population with primary reliance on clean fuels and technology, which is indicator 7.1.2 under the SDG agenda. Using available data from household surveys and national energy balances for these nations, we first assess current patterns in cooking energy demand and recent trends in improving access to clean cooking. We then use the data to calibrate a cooking energy service demand model, which we apply to simulate future pathways of clean cooking uptake. We test different policy mechanisms for encouraging universal clean cooking access in each country by 2030, and quantify the policy costs, health and emissions implications of households’ responses in cooking fuel demand in comparison to a baseline.

## 2 Overview of impacts and barriers

A transition away from the use of solid fuels, like fuelwood, burnt in traditional stoves has important economic, environmental and health benefits. However, several barriers to this transition persist. Access to clean cooking can have benefits for economic development and poverty alleviation [13]. In Central America alone, an estimated 37,000 premature deaths in 2010 were attributable to household air pollution from biomass cooking, of which about 10 percent were children [14]. Strong evidence links exposure to PM2.5 emitted from burning solid fuels in primitive stoves to premature deaths and disability [15,16,17]. The indoor air quality (IAQ) guidelines on household fuel combustion issued recently by the WHO indicate that in order to protect public health, PM2.5 concentration at or below 35 μg/m3 are required [18]. However, field evaluation studies show that most solid fuel cookstoves lead to kitchen PM2.5 concentrations of ten or more times this level. Improving household cooking conditions through the use of advanced stoves, cleaner and more efficient fuels, and better ventilation has the potential to substantially improve public health.

Dependence on solid fuels for cooking also affects climate because inefficient combustion in traditional stoves releases short-lived climate forcers (SLCF), such as methane and black carbon [19,20]. The residential sector is estimated to be the largest energy related source of carbonaceous aerosol, with black carbon ranked as the second largest contributor to global warming, and also known to affect local climate and weather patterns [21,22,23,24]. Recent analysis of the net emissions from household cooking energy transitions in India points to the importance of unsustainable biomass harvesting and SLCF in determining the extent of these impacts [25]. Quantifying the emissions impacts of household energy transitions is thus important to understanding these local and global environmental impacts.

Recent energy policy statements from the Central American governments recognize the challenges faced by the residential cooking energy sector. The “2020 Central American Sustainable Energy Strategy”, coordinated by the United Nations Economic Commission for Latin America (ECLA) focuses on securing the sustainability of the region’s energy supply by reducing the demand for oil‐based products, increasing the supply of renewable energy sources, improving energy efficiency and mitigating the effects of energy use and production on the environment. The strategy also particularly stresses efforts towards increasing access to energy services for isolated and lower‐income populations. All countries in the region are also signatories to the Central American Integration System (Sistema de la Integración Centroamericana, SICA). SICA has identified clean cooking as the region’s high priority in its Sustainable Energy Strategy for 2020 with a goal to reduce fuelwood consumption and install one million efficient stoves by 2020 [26].

Several programs in the past have led to the dissemination of improved biomass cookstoves globally and in the region. A review of some of these for the region specifically indicate that their impact has been mixed, both in terms of numbers of households benefited and the resulting health benefits [27,28,29,30,31]. A recent International Finance Corporation (IFC) study highlights several barriers both for consumers and producers or distributors that are responsible for the limited adoption and scale‐up of clean cooking solutions [32]. The most important among these are issues of awareness, affordability and availability of better cooking alternatives, as well as the lack of capital both for consumers and producers/distributors. Similar factors have also been identified in recent reviews of literature on factors influencing uptake of clean cooking solutions by households [33,34].

Experience suggests that overcoming barriers to the adoption of cleaner stoves, including consumers’ inability to pay and liquidity constraints, requires putting in place appropriate consumer financing strategies [35,36]. When biomass is collected freely, there is little financial incentive for households to buy improved stoves, although the labor required might be significant. In such circumstances, the question is not whether consumer financing or grants are required or not, but rather how these should be designed and targeted. Existing evidence suggests that demand for environmental health technologies in low‐income households is highly price elastic, so that stove price rebates or grants may stimulate household purchase of cleaner stoves [37,38]. Such rebates or grants also need to be supplemented with information and education programs aimed at inducing behavior change and to encourage long‐term adoption and sustained use of cleaner stoves [39]. Accounting for social and cultural preferences is also critical to scaling up the diffusion of cleaner stoves [40].

## 3 Recent trends and current patterns

### 3.1 Survey data sources and macro trends in cooking fuel use

We rely on recent and comprehensive household surveys available to assess current patterns of cooking energy use in the three nations of focus in this study. For details of the household surveys used in this analysis see Table 2. The household survey datasets are further supplemented by energy balances and statistics from the IEA and OLADE, aggregate national data on fuelwood consumption from the Tropi‐ WESTAT database [41], and information on energy prices and costs from the surveys and officially cited sources [42,43,44].

**Table 2:** Household survey data sources and macro trends in residential energy use.

Country	Household survey data source	Residential LPG use (GJ/ per capita)			Residential biomass use (GJ/ per capita)		
		1990	2000	2010	1990	2000	2010
**Guatemala**	Encuesta Nacional de Condiciones de Vida, ENCOVI 2006 [54]	0.38	0.66	0.61	13	13	13
**Honduras**	Encuesta Leña Honduras 2011 [55]	0.11	0.25	0.16	17	14	11
**Nicaragua**	Encuesta Ingresos y Gastos de los Hogares 2006‐2007 [56]	0.08	0.17	0.25	13	11	10

Biomass and LPG are the major cooking fuels used in the region. LPG use for cooking has grown steadily in recent decades in all three nations, but particularly in Nicaragua. However, because rural areas lack supply infrastructure, LPG use is concentrated in cities. Significant transportation and distribution costs, particularly in rural areas, also restrict wider adoption of LPG in these regions. More recently, the substitution of LPG for fuelwood has slowed and even reversed in Guatemala and Honduras (Table 2). This is, in part, a result of the elimination of subsidies. Aside from cost and availability, resistance to switching to LPG is also driven by personal preferences, for example, LPG is considered not suitable for making tortillas [45]. Guatemala and Nicaragua have deregulated all petroleum fuel prices. Guatemala imposes a tax on LPG, while Nicaragua neither taxes nor subsidizes LPG. Honduras regulates the wholesale and retail prices of oil products in the country based on import parity criteria, and has a small subsidy on LPG [44].

The populations of Guatemala, Honduras, and Nicaragua remain highly dependent on fuelwood as a source of energy. In aggregate, residential consumption of biomass has increased steadily in all three countries over the last few decades despite a gradual reduction in the share of population using the fuel. There was a slight decline in the average per capita fuelwood consumption over this period, but the average consumption among fuelwood users remained almost unchanged or even slightly increased in Guatemala and Nicaragua.

There are also some important differences in demographics and socio-economic patterns across the countries. Nicaraguans have particularly large households in urban areas compared to the other countries. Furthermore, although Nicaragua and Honduras have comparable and lower GDPs per capita than that of Guatemala, because of Nicaragua’s high income inequality, the share of urban high income population, earning more than PPP$5 per capita per day (62%) is closer to that of Guatemala (80%), than Honduras (28%). This may have implications for the future uptake of LPG, since this income group typically is the most likely to switch completely away from traditional fuels.

### 3.2 Household cooking fuel use patterns

Though differing in population, economic circumstances, and resource endowments, the three nations studied have fairly similar patterns of household cooking fuel use. Guatemala is the largest of the three nations with a population of over 15 million inhabitants. The residential sector is responsible for over 98 percent of biomass energy consumed in the nation [46]. According to the ENCOVI 2011 survey, over 90 percent of rural households and about half of urban households continue to rely on traditional cooking fuels and stoves [47]. More than a quarter of urban households and about half of rural households collect all the fuelwood they consume, while others collect a portion and purchase the rest [48]. For households that purchase all or a part of the fuelwood they consume, fuel savings could be a significant motivator for adopting improved stoves.

Honduras has just over half the number of inhabitants as Guatemala. In contrast to the other two nations, a fraction of the population in this nation cooks with electricity. Data for 2011/2012 from the Demographic and Health Survey suggests that while biomass dependence is still high among rural households, over two-thirds of urban households rely primarily on either LPG or electricity for cooking [49].

Nicaragua is the poorest of the three Central American nations included in our analysis. In 2010, an estimated 97 percent of the rural population cooked with fuelwood [45]. While the urbanization rate in this nation is higher than in the other two countries, almost half of the urban population also still depends on biomass. The prices and per capita use of fuelwood in cities is lower in Nicaragua than in Honduras and Guatemala (Table 3, Table 4). The low prices may explain the high dependence on fuelwood among the urban population. It may also explain why so many urban non‐poor households continue to use fuelwood instead of gas.

**Table 3:** Average cooking fuel prices, by country and fuel.

Country	Fuelwood		LPG	
	Average Use (National)	Average Price (Urban)	Average Use (Urban)	Average Price (Urban)
	kg/cap/day	$/GJ_ue_	MJ/cap/day	$/GJ_ue_
**Guatemala**	2.83	90.5	1.8	92.4
**Honduras**	2.52	48	4.7*	61.9
**Nicaragua**	2.54	9.5	2.95	67

*Note: LPG use in Honduras imputed. $/GJ_ue_ refers to price per Giga Joule of useful energy.

**Table 4:** Household cooking energy use patterns in Guatemala, Honduras, and Nicaragua.

Classification By Income Group	General Population Characteristics				Gas/ Electricity Users*			Biomass Users (Purchasers)			Biomass Users (Collectors)		
	HH Size	Avg. Income	Pop Share	Cooking	Gas Use	Users	Exp. Share	Bio purch.	Users	Exp. Share	Bio Coll.	Users	Exp. Share
2005PPP$/capita/day	Average	PPP$/cap/day	(%)	Exp. Share	MJ/cap/day	(%)	(%)	MJ/cap/day	(%)	(%)	MJ/cap/day	(%)	(%)
**Rural Guatemala 2006**		** **	** **	** **	** **	** **	** **	** **	** **	** **	** **	** **	** **
0–2	6.4	1.2	26%	7.8%	3.1	4%	1.9%	37.5	32%	8.7%	37.3	68%	0%
2–5	5.7	3.3	34%	4.9%	3.4	14%	0.8%	38.2	43%	4.7%	44.3	55%	0%
>5	4.8	12.0	40%	2.1%	4.0	45%	0.3%	35.7	46%	1.9%	43.6	40%	0%
**Urban Guatemala 2006**		** **	** **	** **	** **	** **	** **	** **	** **	** **	** **	** **	** **
0–2	5.9	1.3	4%	13.5%	3.8	20%	2.0%	28.9	53%	13.5%	38.5	41%	0%
2–5	5.2	3.5	16%	6.9%	3.9	44%	0.8%	31.0	56%	5.2%	36.3	24%	0%
>5	4.2	19.5	80%	1.6%	5.0	87%	0.2%	26.5	27%	0.8%	35.1	8%	0%
**Rural Honduras 2011**	** **	** **	** **	** **	** **	** **	** **	** **	** **	** **	** **	** **	** **
0–2	6.6	1.2	40%		0.8	6%		50.1	41%	6.7%	43.6	53%	0%
2–5	4.7	3.1	43%		4.0	26%		55.3	44%	1.8%	66.0	37%	0%
>5	3.3	10.7	17%		7.6	55%		58.1	33%	1.1%	75.2	16%	0%
**Urban Honduras 2011**		** **	** **	** **	** **	** **	** **	** **	** **	** **	** **	** **	** **
0–2	6.2	1.3	23%		3.5	30%		43.1	51%	6.0%	49.8	22%	0%
2–5	4.6	3.3	48%		6.0	52%		52.6	34%	2.1%	54.6	16%	0%
>5	3.3	9.8	28%		10.7	74%		82.8	29%	1.3%	73.0	7%	0%
**Urban Nicaragua 2006/07**		** **	** **	** **	** **	** **	** **	** **	** **	** **	** **	** **	** **
0–2	8.5	1.6	3%	5.0%	2.1	14%	5.0%	48.1	84%	5.1%		2%	0%
2–5	6.7	3.6	35%	3.6%	3.3	48%	3.3%	43.2	53%	3.6%			
>5	4.9	12.0	62%	1.8%	5.0	78%	1.9%	34.7	22%	1.6%			

Note

*Gas/Electricity column reflects the use of both only in Honduras. Electricity for cooking is not prevalent in Guatemala or in Nicaragua. Biomass use in Nicaragua imputed assuming uniform cooking energy needs. LPG use in Honduras is also imputed.

### 3.3 Commonalities and differences across the nations

Comparing the patterns of cooking energy use across the three countries, we observe some commonalities as mentioned above, but also some distinct differences. In all three countries we observe a growing population in rural areas without viable substitutes for fuelwood, combined with increasing use of LPG in cities. In all three countries, fuelwood consumption still remains substantial in urban areas, though to a greater extent in Nicaragua and Guatemala than in Honduras. On average, per capita fuelwood use is similar across the countries, between 2.5‐2.8 kilograms per capita per day (Table 3). Fuelwood meets a substantial part of cooking needs even in the high income group, between a quarter in Guatemala, and almost a half in Honduras (Table 4). LPG prices are relatively similar across the countries, in comparison to fuelwood price differences (Table 3).

Cooking energy needs differ across and within the countries, despite similar diets. Cooking useful energy demand is highest in Honduras and lowest in Nicaragua reflecting also differences in income levels (see Table 3). Among the urban populations, fuelwood prices differ significantly–Guatemalans pay almost twice the price as Hondurans, while Nicaraguans pay an order of magnitude less than Guatemalans. This is reflected in their corresponding cooking budgets, which points to the inelasticity of energy use to fuel prices. Among urban households, the share of LPG that meets total cooking energy demand is highest in Guatemala, followed by Nicaragua and Honduras, which is consistent with the population share in the highest income group in the three countries (Table 4).

In the following we build on these insights and employ the data from the household surveys to calibrate a model of household cooking energy choices and demand that is then used to assess future policy scenarios for increasing access to clean cooking in the region by 2030.

## 4 Methods, model and scenarios

In this study we rely on the MESSAGE-Access model, a residential cooking energy choice and demand model that that has been applied earlier to South Asia [50]. A schematic overview of the data sources and model are presented in S1 Fig in the Supplementary Information. The model employs data on fuel use and expenditures for cooking to estimate fuel demand curves that are then employed to construct and assess future policy scenarios for clean cooking access in terms of costs, impacts on health, and GHG emissions. For the current period, cooking demands, expenditures, and household characteristics are calibrated using data derived from the representative household surveys introduced in the previous section.

In the model, population is grouped by income and urban/rural status to represent heterogeneities in the affordability and availability of cooking fuels. We choose income divisions to represent significant poverty benchmarks but also to maintain approximately even population between groups in the start year of the model. The divisions are defined as per capita daily total expenditure in 2005 PPP$ below $2/day, $2-$5/day, and over $5/day for rural households and under $5/day and over $5/day for urban households. For future scenarios, population and income projections for each nation are drawn from the Global Energy Assessment (GEA) database. Here, we use the GEA’s medium scenario (GEA‐M) as a source of population and income data and projections (see S1 and S2 Tables). For fuel price projections we use the GEA‐M forecasted electricity and LPG prices (see S3 Table). To represent future changes in household size, cooking energy demand, and cooking expenditure, we estimate the relationship of these variables as a function of household income using the data from the household surveys for each nation.

To estimate cooking fuel demand in future years, fuel-specific demand curves are estimated for each household group by regressing the quantity of fuel purchased on the cost to cook with that fuel. Demand curves are estimated directly from the household survey data applying a best-fit power function that assumes price elasticities are constant over time, but distinct for each household group. The demand curve equation takes a form of Eq (1):

$$D_{e}^{f}=a_{e}^{f}\times {\left(C_{e}^{f}\right)}^{b_{e}^{f}}\times S_{e}^{f}$$ (1)

Where ***e*** is expenditure group, ***f*** is fuel type, ***D*** is demand for useful cooking energy, ***C*** is cooking cost per unit useful energy including stove and fuel, ***S*** is share of total cooking energy demand met by fuel ***f***, and ***a*** and ***b*** are coefficients. We present an example demand curve in S2 Fig.

Cooking cost in the model is determined as a function of not only the fuel price, but also the price of stoves. To incorporate stove costs into the overall cooking cost, stove prices are amortized over the total useful energy delivered, accounting for differences in the efficiencies of different stove-fuel combinations (see S4 Table for assumed stove costs and characteristics). Fuel prices for the three modeled fuels were derived from household surveys when available by taking the weighted mean of reported fuel expense divided by reported fuel use. Where fuel price data was not available from household surveys, we used officially cited sources [43, 44, 45]. The stove cost is amortized using a discount rate unique to each population sub‐group calculated as a function of total household income.

To model how consumers choose between fuels, we assume “fuel-preference tiers” based on evidence that households ascend a metaphorical “energy-ladder” as they get richer [51]. LPG and electric induction that are predominantly chosen and used by more affluent household groups are assumed to be the top-choice (Tier 1) fuel-stove options for all consumers. We assume the service of these two fuel-stove combinations is equally desirable and therefore assume the cheaper of the two Tier 1 fuels will be chosen in each period. We then assign improved biomass cook-stoves (ICS) as a Tier 2 cooking system and traditional (three-stone) biomass stoves as Tier 3, the fallback option using free, collected, firewood. The defined tiers determine the sequence in which the estimated demand curves are deployed to meet a fixed cooking energy demand in response to a set of fuel prices in a given year. Cooking energy demands change with time as a function of household size and income. We account for these changes by regressing income against household size, expenditure on cooking, and cooking energy demand using the cross-sectional data from the household surveys, and employing the income and population projections from GEA-M.

Of the three countries analyzed in this study, only the Guatemala survey provides sufficient data to derive demand curves directly from the survey. It includes both fuel demand and price for fuelwood and LPG for a nationally representative sample of the population. The survey, however, provides no information on the type of stove used for cooking with fuelwood. The demand for improved biomass cookstoves was, in this case, estimated by using data on fuelwood purchases as a proxy for household willingness‐to‐pay for the improved stoves. We, therefore, only estimate demand for the improved stoves among households purchasing fuelwood and the expenditure on fuelwood is used as an indicator of the amount households were willing to spend on cooking with this fuel. The demand curves estimates from Guatemala are adapted in conjunction with information from the household surveys from Honduras and Nicaragua to develop independent demand models for each country. For Nicaragua and Honduras, we adjust the demand curves using those estimated from the household survey for Guatemala so as to reflect actual demand and cooking costs in each of the three nations. This is done by adjusting the estimated coefficients of Eq (1). To do so cooking demand (**D**) and cooking cost (**C**) are estimated from the surveys of each country for each household group. For Nicaragua and Honduras coefficient **b** is set equal to that of the corresponding household group in Guatemala, and **a** is determined using Eq (2):

$$a_{e}^{f}=\frac{D_{e}^{f}}{{C_{e}^{f}}^{b_{e}^{f}}}$$ (2)

In order to evaluate alternative future transition scenarios and their effectiveness in accelerating a transition to better cooking fuels and/or stoves, we construct a set of distinct future pathways as described in Table 5. The transition scenarios are constructed to assess the impacts of fuel price support policies on cleaner fuels like LPG (these are prefixed by T–tariff or TT–targeted tariff in scenario names) and/or the effectiveness of grants/ rebates or microfinance credit (referred to by the prefix G–grant or TG–targeted grant in scenario names) to reduce the initial investment or lower upfront costs associated with the purchase of better stoves (see S5 Table for model R code). In the case of targeted scenarios, the beneficiaries are all households in rural regions and households spending less than PPP$5/capita/day in urban areas. The grants policies for stoves range from moderate at 50 percent to aggressive at 100 percent rebates on the stove price. The grants or rebates scenarios for improved biomass stoves (G_ICS) and electric stoves (G_EIS) are applied universally, whereas for LPG stoves these are targeted to rural households and households spending less than PPP$5 per capita per day in urban areas (TG_LPG). Three alternative scenarios for targeted fuel price or subsidy support to LPG fuel for cooking are also explored, which are also targeted to the same population segments (TT_LPG). The LPG fuel price support scenarios include those that provide a 20%, 30% and 50% subsidy level.

**Table 5:** Policy scenarios for assessing future household cooking transitions. Note that all targeted* policies are for all rural and <PPP$5/capita/day households in urban areas.

No.	Scenario Name	Description
**1**	NNP	No new policies (a business-as-usual scenario)
**2**	G_ICS_50	Grant or rebate on improved biomass stoves, @50%
**3**	G_ICS_100	Grant or rebate on improved biomass stoves, @100%
**4**	TG_LPG_50	Targeted* Grant or rebate on LPG stoves, @50%
**5**	TG_LPG_100	Targeted* Grant or rebate on LPG stoves, @100%
**6**	G_EIS_50	Grant or rebate on electric induction stoves, @50%
**7**	G_EIS_100	Grant or rebate on electric induction stoves, @100%
**8**	TT_LPG_20	Targeted* Transfer for Fuel Price Support on LPG, @20%
**9**	TT_LPG_30	Targeted* Transfer for Fuel Price Support on LPG, @30%
**10**	TT_LPG_50	Targeted* Transfer for Fuel Price Support on LPG, @50%

In our baseline scenario (NNP), no new access policies are assumed, but a gradual shift to clean cooking occurs as clean cooking becomes more affordable with rising incomes and growing urbanization in all three countries. However, growth in LPG penetration is constrained by the actual preferences observed in the household surveys that also reflect non‐monetary constraints on cooking choices, which we capture in the demand curve. The constraints and preferences are reflected in the lower share of LPG use in rural as compared to urban households with similar income levels (see Table 4). This, in some instances, might be because of a lack of distribution and access to fuels like LPG in rural areas, but can also reflect habits, preferences and tastes (as for example for tortillas cooked on wood-fired stoves).

We run the model every decade, assuming market conditions stay constant within the decade (for e.g., 2021–2030). We estimate annual policy costs for each policy for 2020 and 2030 and cumulative policy costs over the period from 2020 to 2030. The annual cost of policies in a given year is calculated as the number of stoves (in the case of a grant policy) or volume of fuel purchased (in the case of a tariff policy) in each year multiplied by the annualized stove cost or fuel cost subsidized in each year, all in 2010 US dollars. We also estimate policy cost per beneficiary by dividing annual policy cost by the number of persons “migrated” by the policy. In the case of ICS grants, “people migrated” refers to those who use traditional three‐stone stoves in 2030 under the NNP scenario, but can afford an ICS stove in 2030 with the policy support. In the case of LPG, “people migrated” refers to those formerly using either three‐stone or ICS in 2030 under the NNP scenario.

We estimate emissions impacts at the point‐of‐use using standard IPCC emission factors. We do not include upstream emissions. Little information is available to determine how much of the biomass used for cooking in the region is renewably harvested. This information is crucial for determining the CO2 emissions from burning fuelwood. One regional estimate of sustainable fuelwood, defined as the wood harvested or collected that does not contribute to loss or degradation, is cited at 60 percent [45]. The most recent and comprehensive analysis of the contribution to global forest loss of fuelwood harvesting for meeting cooking demands concludes that about 27 to 34 percent of wood fuel harvested worldwide would be considered non‐renewable [52]. According to the assessment, this is estimated based on whether or not annual harvesting exceeds incremental re‐growth. For the year 2009, estimates of the fraction of non‐renewable biomass extraction from the study for Honduras and Nicaragua are significantly higher than the global average. For Guatemala this is estimated at 32 to 35 percent, for Honduras 64 percent, and for Nicaragua 58 percent [52]. We use a conservative estimate of 35% in our analysis.

To estimate the health impacts of solid fuel use we use the methodology developed by the Global Burden of Disease comparative risk assessment [14]. We employ the latest relative risk estimates for diseases associated with exposure to pollution from solid fuel combustion [16], and use the population dependent on solid fuels as an exposure surrogate.

## 5 Results of scenarios to accelerate clean cooking access

### 5.1 Baseline outlook with no new policy (NNP)

**Fig 1:**
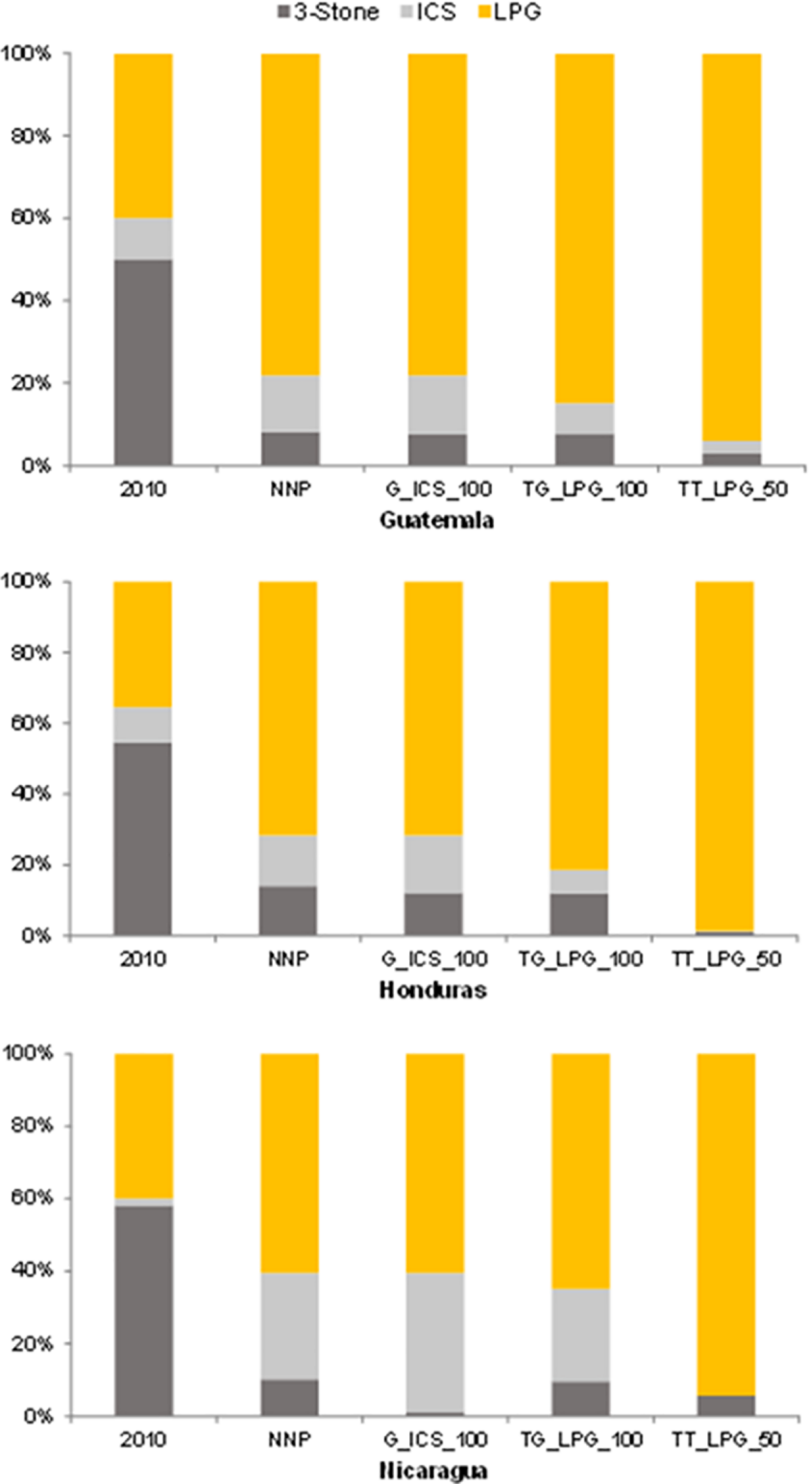
Stove shares in 2010 and under alternative policy scenarios in 2030.

Under our baseline scenario, about half of rural Guatemalans are unable to afford clean fuels or stoves in 2030. However, in urban Guatemala only 5 percent of the population is likely to be unable to afford clean fuels or stoves. In Honduras, in urban areas, over three quarters of the population is likely to be able to afford clean fuels or stoves by 2030. Among rural households, however, about two‐thirds will be unable to afford clean fuels or stoves in 2030. In urban areas of Nicaragua, only 6 percent of the population is likely to be unable to afford clean fuels or stoves by 2030. Among rural households, however, about three quarters will be unable to afford clean fuels or stoves in 2030 (see Fig 1).

### 5.2 Policy scenarios to accelerate access

A fuel subsidy of 50% on LPG could bring solid fuel use down to 5 percent by 2030, and benefit the poorest two-fifths of the population in all three countries (Fig 1). The implementation of different policies alters both the shares of different stoves in use as well as the total achievement of clean cooking access. A capital grant for the purchase of improved stoves has little impact in Guatemala, where fuelwood costs are so high that reduced stove costs do not significantly change the total cost of cooking with improved stoves. Similarly, as LPG prices are highest in Guatemala, stove costs have a relatively low share in total cooking costs and stove grants for LPG stoves are likely to shift only 1.1 million people to the use of LPG by 2030 (Table 6). While the cost of a fuel price support policy on LPG of 50% is significantly more expensive at an estimated cost of US2010$47.8 per beneficiary, it could benefit almost 2.7 million Guatemalans.

**Table 6:** Population benefited by and costs per beneficiary of alternative policy scenarios.

	G_ICS_100	TG_LPG_100	TT_LPG_50
**Guatemala**			
**Population Migrated (mil)**	2.33	1.15	2.68
**Cost per beneficiary (US_2010_$/year)**	1.10	5.93	47.80
**Honduras**			
**Population Migrated (mil)**	1.58	0.84	2.81
**Cost per beneficiary (US_2010_$/year)**	0.93	6.32	34.99
**Nicaragua**			
**Population Migrated (mil)**	2.18	0.27	1.85
**Cost per beneficiary (US_2010_$/year)**	0.64	3.18	13.39

As in the case of Guatemala, the 50% LPG fuel subsidy support scenario is the most effective of the set of scenarios we implemented for enabling a transition to clean cooking fuels in Honduras by 2030 (Fig 1). The policy, targeted to rural households and urban households living below PPP$5/capita/day, would benefit the bottom 60 percent of the population in the country. Such a policy could make clean fuels affordable almost universally (to about 98 percent of the population) by 2030. A scenario in which a rebate or grant is provided for the use of electric induction cookstoves in the region is also somewhat effective only in Honduras, which is the only country out of the three analyzed that currently uses electricity for cooking due to its low electricity prices. Such a capital subsidy on electric stoves could benefit up to 45 percent of the population in 2030 (see S6 Table for additional results). However, a more aggressive increase in electricity prices in the region or slow growth in installed power capacity would make such a transition less feasible.

Under the 50% LPG fuel subsidy support scenario, about 2.8 million people can move off solid fuel use in Honduras by 2030. A grant or rebate policy on improved biomass stoves is also reasonably effective here, since fuelwood prices are not as high as in Guatemala. Such a policy could benefit 1.6 million Hondurans in 2030 and could cost below US2010$1 per beneficiary (Table 6). As a practical matter, stove grant policies, if combined with a massive scale‐up of improved cook stoves, might be a cost‐effective interim solution in Honduras, where for many rural households a shift away from biomass may be out of reach in the short term. In Honduras, LPG stove grants are also relatively more effective as compared to Guatemala.

Of the three countries, fuelwood prices are the lowest in Nicaragua. As a result, the total levelized cost of three‐stone and improved cook stoves are very close even without the stove grants. Therefore, a grant or rebate on improved stoves is very effective in bringing about a transition to the use of improved biomass stoves in Nicaragua. Such a policy could benefit 2.2 million of the population and is estimated to cost US2010$0.64 per beneficiary (Table 6). The costs of a fuel price support policy on LPG of 50% while more expensive at an estimated cost of US2010$13.4 per beneficiary, could benefit 1.85 million Nicaraguans.

### 5.3 Implications for cooking energy demand, climate emissions, and human health

**Fig 2:**
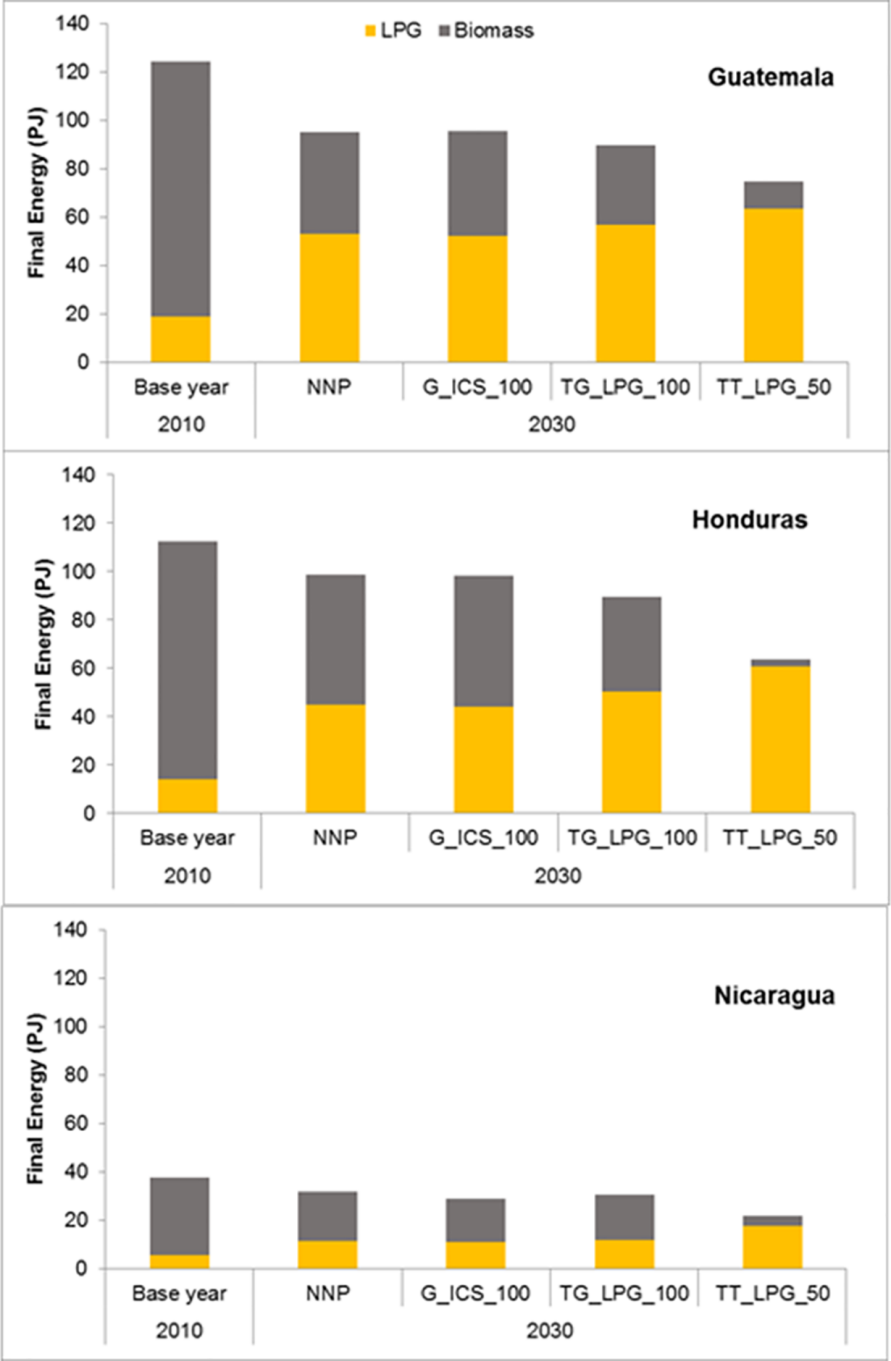
Final energy for cooking in 2010 and 2030 under alternative policy scenarios.

The impact of alternative transition scenarios for improving access to clean cooking on cooking energy requirements is depicted in Fig 2. With population growth, cooking energy demand is expected to increase over time. However, due to the shift to more efficient stoves (traditional stoves are approximately 15 percent efficient in comparison to approximately 25 percent for improved stoves and 60 percent for LPG stoves) even in the absence of policies, final energy demand is likely to be lower by 18 percent by 2030. Under the 50% fuel price support on LPG scenario, final energy use could be 29 percent lower than the no new policies scenario. These reductions are similar in all three countries. However, the relative share of the reduction in each country from income growth and policy implementation differ, and depend on each scenarios effectiveness in increasing clean cooking access and also by the penetration of LPG relative to that of improved stoves. Thus, in Honduras, energy demand reduction from a rapid transition to LPG is the highest, followed by Nicaragua and then Guatemala.

**Fig 3:**
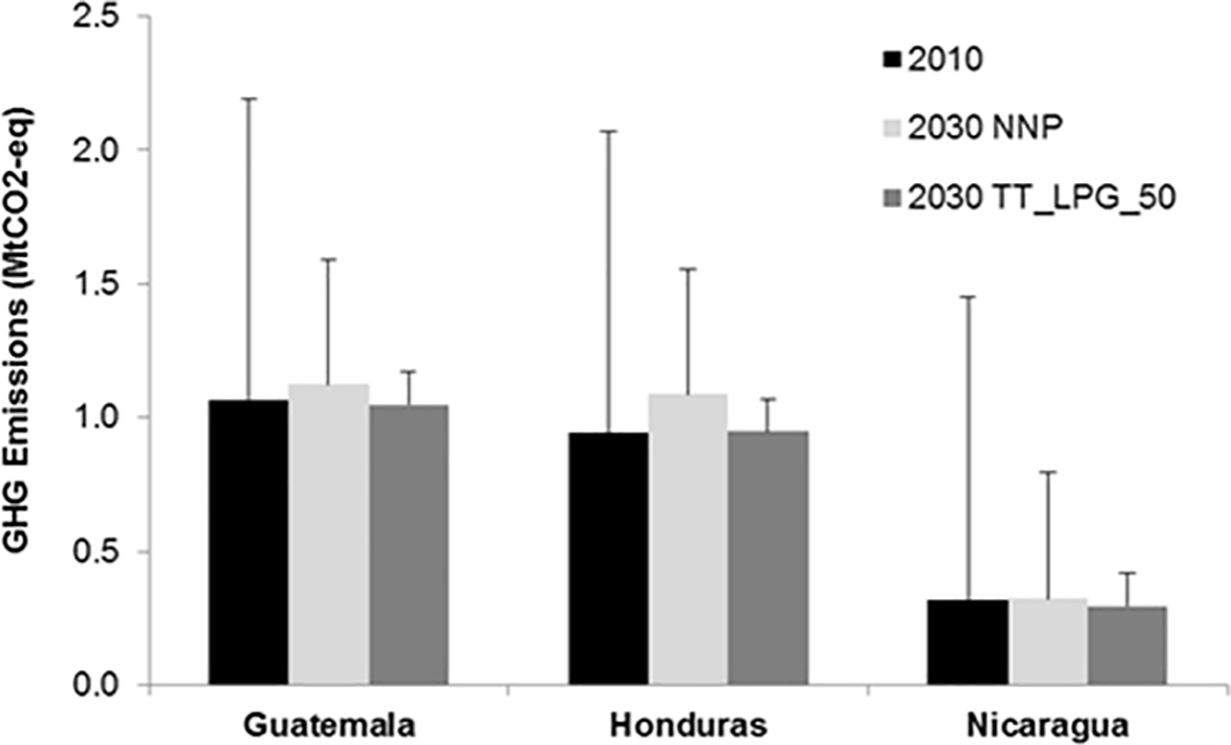
GHG emissions in 2010 and 2030 under the NNP and TT_LPG_50 scenarios.

Fig 3 presents the impacts of alternate transition scenarios on total GHG emissions for 2010 and 2030. Compared with the year 2010, GHG emissions in 2030 are projected to increase by 9 percent (collectively in the three countries) without any policies, but would be 1 percent lower in the 50% fuel price support for LPG scenario. Most of the increase in emissions is accounted for by the additional LPG demand for cooking. However, the net impacts of the access policy are marginally favorable due to the efficiency improvements associated with LPG use in place of biomass.

In the analysis presented in Fig 3, the error bars depict the additional CO2 emissions that would result under the assumption that 35 percent of biomass used for cooking in these countries is not renewably harvested. The assumption of 35 percent as the fraction of non‐renewable biomass (fNRB) in the region is conservative given estimates of the range of values for a sub‐set of least developed countries as calculated for the Kyoto Protocol’s Clean Development Mechanism (CDM) and carbon offset projects (CDM 2012), and as noted for the region in Bailis et al. (2015). Accounting for the CO2 emissions from non‐renewable biomass use suggests that the emissions impacts of achieving universal clean cooking access in the region could be even more favorable for the climate. Emissions in this case could be 35 percent lower than in the baseline in 2030.

**Fig 4:**
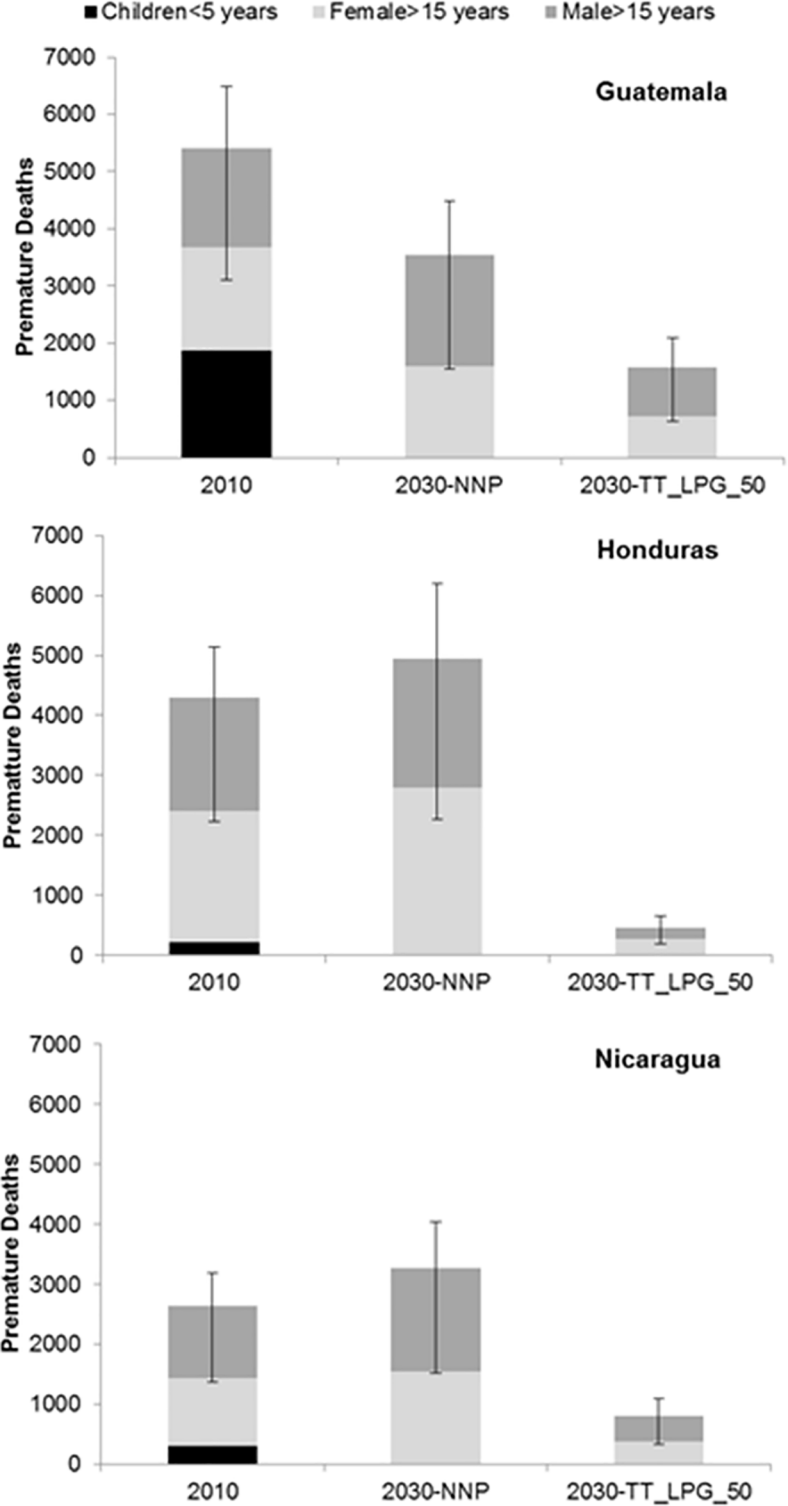
Premature deaths attributable to household air pollution from solid fuel use in 2010 and 2030 under the NNP and TT_LPG_50 scenarios.

We include only Kyoto gases in our estimate of GHG emissions impacts of alternative policy scenarios. However, the use of solid fuels in traditional cooking stoves, also results in emissions of short lived climate forcers (SLCF) like black carbon that also has significant environmental, health and economic costs. Exposure to particulate matter from household cooking in fact remains a major cause of death and disability in these three countries. In 2010, total deaths attributed to solid fuel combustion in traditional stoves in the three countries studied amounted to 12,353 (with a confidence interval of 6,709‐14,804, for low and high relative risk rates ‐ RR), with the impacts felt mainly by women and children (Fig 4). An almost equivalent number of deaths, about 11,740 are estimated in 2030, in the NNP scenario. Although there is uncertainty associated with these estimates, policies that achieve universal access to clean cooking by 2030 have the potential to avert about 8,890 (Low RR: 4,193, High RR: 10,909) premature deaths in 2030. In other words, in the absence of any new policies to enhance access to modern cooking fuels or devices, it is estimated that in 2030 almost an equivalent number of lives will be lost due to household air pollution, compared to estimates for 2010. Deaths attributable to ALRI among children under 5 are seen to decline between 2010 and 2030 even in the absence of any access policies, but deaths due to chronic obstructive pulmonary disease (COPD), ischemic heart disease (IHD) and strokes in adults are expected to increase during the same period. These trends are in line with those reported by others (for e.g.,[53]), who find that the observed decline in childhood ALRI mortality over time is a result of an improvement in other factors reducing the risk of this communicable disease, whereas the upward trend in adult incidence of non‐communicable diseases is mainly due to an increasing ageing population share, particularly in Honduras and Nicaragua.

## 6 Conclusion and lessons for a clean cooking outlook by 2030

Reducing fuelwood use for cooking in Central America is an important policy imperative. With rising incomes, over 90 percent of urban residents, some of whom currently use biomass in Guatemala and Nicaragua, are likely to shift to clean fuels without any policy change. However, in urban Honduras, where per capita income is projected to be lower, about a quarter of the urban population could still be relying on traditional fuelwood stoves for cooking in 2030. In rural areas, solid fuel dependence is likely to be much higher. Without supporting policies, between 40 to 50 percent of rural Guatemalans and Hondurans, and over two-thirds of the population in rural Nicaragua, are unlikely to find clean fuels or stoves affordable in 2030. In 2010, over twelve thousand premature deaths were estimated to be attributed to traditional stove use. This number could remain almost unchanged by 2030 in the absence of new policies. From a climate perspective, GHG emissions from cooking, from population and income growth, are estimated to see a modest increase (~9%) from 2010 levels, since more efficient stoves and fuels like LPG will replace inefficient biomass. If some extent of existing biomass use is assumed to be non‐renewably harvested, then even in the absence of any policies, emissions might be expected to decrease due to displacement of the high CO2 emissions from unsustainable biomass harvesting. However, low confidence in existing estimates of fraction of non-renewable biomass is a cause of relatively large uncertainty in estimates of GHG emissions from biomass as compared to LPG.

Here we have examined a set of cooking energy transition scenarios for a shift to LPG and improved cookstoves to assess some key impacts of achieving universal access in the three countries by 2030. We find that of the scenarios we studied, a 50% fuel tariff support policy for LPG is most effective in enabling a transition to clean cooking. Such a transition would need to be targeted to the rural and poor urban populations. By 2030, such a transition scenario could make cooking with LPG affordable to an additional 7.3 million people in these three countries. While the policy costs of such a subsidy policy are estimated to be higher compared to stove grant policies, it is more effective in shifting the population to clean cooking by 2030 in all three countries and achieving the universal access goal. Also, under this scenario 8,890 premature deaths could be prevented in 2030. For the three countries together, the costs of such a fuel price support for LPG would be US$251 million/year in 2010 prices. The cost per beneficiary is estimated to be as low as US$13/year in Nicaragua to as high as US$48/year in Guatemala. The GHG impact is at worst marginal, assuming (conservatively) that all biomass is renewably harvested. However, if even 35 percent were not renewably harvested, which is far less than what literature suggests, the 50% subsidy on LPG scenario could lead to a 35 percent reduction in GHG emissions relative to the baseline in 2030. A policy of grants or microfinance loans for improved biomass cookstoves purchases could be an effective interim solution for certain regions, particularly in Nicaragua, where fuelwood is purchased and prices are low, so that stove costs constitute a much larger share of total cooking costs for the population. Such a policy would cost US$1 million per year and could benefit up to 2.2 million people relative to the baseline in 2030.

Recent policy initiatives and government pledges in these countries have targeted a scaling up of efforts to disseminate improved biomass cookstoves in the region. These targets are on par with feasible transitions, when grants or microfinance for the stoves are made available. In the case of Nicaragua, the targets set (40,000 stoves per year) might be conservative, as a much more rapid uptake of the improved cookstoves may be feasible according to this analysis. However, achieving these more ambitious goals would require significant institution building and strengthening to enable the massive scale‐up in distribution of improved stoves this would entail.

Central American countries have been deregulating LPG markets over the last decade. Future policies aimed at making cleaner fuels like LPG more affordable will need to focus on better design and implementation for targeting subsidies or consider cash transfers and other social service delivery mechanisms for the poor as an alternative. This will require additional capacity building to strengthen the administration of governance systems and local institutions in the region. It is clear that despite the potential benefits, enabling clean cooking access in the region will not be easy. It will demand mobilizing additional financing, ensuring technologies deployed are affordable and acceptable to local communities, and that local capacity is developed to ensure efforts are sustainable in the long term.

There are some caveats to the results presented here. In our analysis and model we do not capture the administrative and implementation costs of policies. Nor are we able to capture the political context in each of these countries that may require the use of policy instruments other those explored here. We also include a limited set of stove options in our model that reflect the most common in use currently. The actual cooking solutions selected will ultimately need to be context specific and suited. Despite these inherent uncertainties, our analysis highlights the scale of effort required for achieving SDG target 7.1 and the significant benefits of this for the region.

## Supporting information

S1 Table Population projection in millions by expenditure group. (DOCX)

S2 Table Income projection by expenditure group in 2010$ per capita. (DOCX)

S3 Table Fuel price trajectory by country (2010 $/GJFE–Giga Joule of final energy). (DOCX)

S4 Table Stove costs and attributes. (DOCX)

S5 Table R code for household cooking demand model and analysis. (DOCX)

S6 Table Additional scenario results on share of population using different stoves. (DOCX)

S1 Fig Schematic overview of model and data sources. (PDF)

S2 Fig Example demand curve for LPG in Guatemala for the U2 expenditure group. (PDF)
