# Supplementary material for: Outlook for modern cooking energy access in Central America
Source: PLoS One. 2018 Jun 8;13(6):e0197974. doi: 10.1371/journal.pone.0197974 (PMC5993280; doi:10.1371/journal.pone.0197974)
Supplement: S1 Table — (DOCX) [file pone.0197974.s001.docx]

Table S1: Population projection in millions by expenditure group

| **Income Group** | **Guatemala** | | | **Honduras** | | | **Nicaragua** | | |
| --- | --- | --- | --- | --- | --- | --- | --- | --- | --- |
|  | **2010** | **2020** | **2030** | **2010** | **2020** | **2030** | **2010** | **2020** | **2030** |
| R1 | 1.781 | 0.991 | 0.409 | 1.5 | 0.905 | 0.244 | 0.736 | 0.441 | 0.115 |
| R2 | 2.428 | 2.041 | 1.334 | 1.65 | 1.513 | 0.923 | 0.973 | 0.806 | 0.461 |
| R3 | 3.056 | 5.101 | 6.799 | 0.745 | 1.729 | 2.995 | 0.776 | 1.356 | 2.081 |
| U1 | 1.447 | 1.168 | 0.788 | 2.638 | 2.757 | 2.128 | 1.265 | 1.003 | 0.377 |
| U2 | 5.664 | 8.79 | 12.362 | 1.082 | 2.232 | 4.202 | 2.072 | 3.076 | 4.353 |
