# Supplementary material for: Outlook for modern cooking energy access in Central America
Source: PLoS One. 2018 Jun 8;13(6):e0197974. doi: 10.1371/journal.pone.0197974 (PMC5993280; doi:10.1371/journal.pone.0197974)
Supplement: S2 Table — (DOCX) [file pone.0197974.s002.docx]

Table S2: Income projection by expenditure group in 2010$ per capita

| **Income Group** | **Guatemala** | | | **Honduras** | | | **Nicaragua** | | |
| --- | --- | --- | --- | --- | --- | --- | --- | --- | --- |
|  | **2010** | **2020** | **2030** | **2010** | **2020** | **2030** | **2010** | **2020** | **2030** |
| R1 | 217.98 | 217.98 | 217.98 | 254.53 | 254.53 | 254.53 | 211.98 | 211.98 | 211.98 |
| R2 | 609.11 | 609.11 | 609.11 | 659.62 | 659.62 | 659.62 | 549.35 | 549.35 | 549.35 |
| R3 | 2360.29 | 3273.78 | 5042.72 | 2327.74 | 2682.98 | 3913.31 | 1337.88 | 1793.26 | 2777.21 |
| U1 | 566.04 | 566.04 | 566.04 | 555.78 | 555.78 | 555.78 | 685.29 | 685.29 | 685.29 |
| U2 | 3694.38 | 5351 | 7900.27 | 2110.61 | 2550.13 | 3499.44 | 2388.43 | 3177.22 | 4967.63 |
