# Supplementary material for: Outlook for modern cooking energy access in Central America
Source: PLoS One. 2018 Jun 8;13(6):e0197974. doi: 10.1371/journal.pone.0197974 (PMC5993280; doi:10.1371/journal.pone.0197974)
Supplement: S3 Table — (DOCX) [file pone.0197974.s003.docx]

Table S3: Fuel price trajectory by country (2010 $/GJFE – Giga Joule of Final Energy)

|  | | **2010** | **2020** | **2030** |
| --- | --- | --- | --- | --- |
| **Guatemala** | **LPG** | 28.11 | 29.23 | 30.32 |
|  | **Electricity** | 62.34 | 63.09 | 64.28 |
| **Honduras** | **LPG** | 20.79 | 22.02 | 23.23 |
|  | **Electricity** | 28.25 | 29.08 | 30.38 |
| **Nicaragua** | **LPG** | 21.2 | 22.36 | 23.5 |
|  | **Electricity** | 32.26 | 33.05 | 34.28 |
