# Supplementary material for: Outlook for modern cooking energy access in Central America
Source: PLoS One. 2018 Jun 8;13(6):e0197974. doi: 10.1371/journal.pone.0197974 (PMC5993280; doi:10.1371/journal.pone.0197974)
Supplement: S4 Table — (DOCX) [file pone.0197974.s004.docx]

Table S4: Stove costs and attributes

| **Stove Type** | **Efficiency** | **Lifetime (years)** | **Price (2010 $)** |
| --- | --- | --- | --- |
| **Three Stone** | 0.15 | 3 | 0 |
| **ICS** | 0.25 | 10 | 50.73 |
| **LPG** | 0.6 | 15 | 83.68 |
| **Electric** | 0.75 | 15 | 104.28 |
