# Supplementary material for: Outlook for modern cooking energy access in Central America
Source: PLoS One. 2018 Jun 8;13(6):e0197974. doi: 10.1371/journal.pone.0197974 (PMC5993280; doi:10.1371/journal.pone.0197974)
Supplement: S5 Table — (DOCX) [file pone.0197974.s005.docx]

Table S5: R code for household cooking demand model and analysis

| *#To use the model, modify appropriately for user directory and input data.*  *#Key input data files to run this code for Guatemala have been included at the end of the code*  *#For a public version of the model with input data files for another region visit -*  *#*[*http://data.ene.iiasa.ac.at/MESSAGE-Access/*](http://data.ene.iiasa.ac.at/MESSAGE-Access/)  #############################################################################  *############################ Sets working folder and load data ############################*  # Set the filepath to the appropriate local directory containing program files  setwd(' ')  # Load Survey Data and Parameters  filepath <- ('P:/ene.model/Access_ Guatemala/')  load(paste(filepath,'lam/data/upds/R/Guatemala_Survey2.RData',sep=''))  ############################################################################  *############################## Define scenario* #############################  dem <- 'geam_base'  scen <- 'geam'  id <- 'nnp_0'  coreid <- 'nnp'  itr <- 0  ps <- 0  ca <- 0  cainbms<-30  cagasst<-30  ss<-0.5  pslpgg <- 0  pselec <- 0  pslpggpcts <- ('0')  pslpgggrps <- ('R1,R2,R3,U1,U2,U3')  pslpggpcts <- ('90,90,90,90,90,90')  ##############################################################################  *########################## Define parameters*# ############################  MERtoPPP<-7.63394417/3.05  years<-as.character(c(seq(1990,2005,5),seq(2010,2110,10)))  new_demand_var <- c('a-c','b-c','c-c','d-c','A-c','B-c','C-c','D-c')  new_dmd_array <- array(NA, dim=c(length(new_demand_var),length(years)),  dimnames=list('variables'=new_demand_var,'years'=years))  ###############################################################################  *################## Get fuel price data from file* ####################  ### function to adjust data into an usable format##############  adj_df <- function(df_data)  { rownames(df_data) <- df_data[,1]  { if (ncol(df_data)==2) { df_data <- data.frame(df_data[,-1,drop=FALSE]) }  else { df_data <- data.frame(df_data[,-1]) } }  colnames(df_data) <- gsub('X','',colnames(df_data))  df_data }  ### function to read in fuel prices from tab files  message_fuel_prices <- function(scenario, coreident, iteration)  { if (iteration>=0) { filename <- paste(filepath,'glb/res/regres/fuel_prices.tab',sep='') }  if (iteration< 0) { filename <- paste(filepath,'glb/res/regres/fuel_prices.tab',sep='') }  skip <- 6 # this number determines what row of the cin file output R starts reading - must adjust if number of fuels is changed  nrows <- 6 # this number determines how many lines are read after the starting point defined above - must adjust the number of fuels is changed  result <- read.csv(filename, header=TRUE, sep='', skip=skip, nrows=nrows)  result <- adj_df(result)  result }  ### define years  access_years <- as.character(c(2005,seq(2010,2100,10)))  new_years <- as.character(seq(2010,2100,10))  policy_years <- as.character(seq(2020,2100,10))  avg_years <- as.character(seq(2030,2090,10))  ############################ Guatemala Prices ###############################  prices_2005 <- adj_df(read.table(paste(filepath,'lam/data/upds/R/Guatemala/2005/fuel_price_2005.txt',sep=''), header=TRUE, sep='\t'))  itr_0_prices <- message_fuel_prices(scen,coreid,0)  itr_0_prices <- itr_0_prices*MERtoPPP  adj_2010 <- prices_2005[,'fuel_price']-itr_0_prices[,'2020']  MSG_prices <- itr_0_prices+adj_2010  MSG_prices[MSG_prices<0] <- 0  MSG_prices[,'2005'] <- prices_2005[,'fuel_price']  MSG_prices[,'2010'] <- prices_2005[,'fuel_price']  #############################################################################  ######## *Create an array with fields and variables needed to calculate demands #############*  exp_grp <- c('R1','R2','R3','U1','U2')  stoves <- c('frwd_three','frwd_inbms','lpgg_gasst','elec_elest')  adv_stv <- c('frwd_inbms','lpgg_gasst','elec_elest')  thr_stv <- c('frwd_three')  all_stv <- c('frwd_three','frwd_inbms','lpgg_gasst')  stv_eff <- c('frwd_three'=0.15,'frwd_inbms'=0.24,'lpgg_gasst'=0.60,'elec_elest'=0.75)  variables <- c('exp_ppp_pc','exp_ppp_hh','disc_rate','hh_size','ck_dmd','frwd_three','population','hh_num','cook_ratio','cook_exp')  stv_var <- c('lfyr','an_stv','fuel_price','cook_price')  pp_var <- c('pp_diff','pp_ratio')  dmd_var <- c('orig_dmd','demand_UE')  fuels <- c('frwd','lpgg','elec')  years <- as.character(c(2005,seq(2010,2100,10)))  coeff <- c('a','b')  units <- c('GJ','GJ/cap','GWyr','GWyr_FE')  tier1 <- c('lpgg_gasst','elec_elest')  tier2 <- c('frwd_inbms')  ### dynamic groups ###  dyn_grp <- c('R3'='exp_grpR20','U2'='exp_grpU20')  grp_array <- array(NA, dim=c(length(exp_grp),length(variables),length(years)),dimnames=list('exp_grp'=exp_grp,'variables'=variables,'years'=years))  stv_array <- array(NA, dim=c(length(exp_grp),length(stoves),length(stv_var),length(years)),dimnames=list('exp_grp'=exp_grp,'stoves'=stoves,'variables'=stv_var,'years'=years))  fuel_adj_array <- array(NA, dim=c(length(exp_grp),length(fuels)),dimnames=list('exp_grp'=exp_grp,'fuels'=fuels))  stv_coeff_array <- array(NA, dim=c(length(exp_grp),length(adv_stv),length(coeff),length(years)),dimnames=list('exp_grp'=exp_grp,'stoves'=adv_stv,'coeff'=coeff,'years'=years))  demand_array <- array(NA, dim=c(length(exp_grp),length(stoves),length(dmd_var),length(years)),dimnames=list('exp_grp'=exp_grp,'stoves'=stoves,'variables'=dmd_var,'years'=years))  MESSAGE_demand <- array(NA, dim=c(length(exp_grp),length(stoves),length(years),length(units)),dimnames=list('exp_grp'=exp_grp,'stoves'=stoves,'years'=years,'units'=units))  constraint_array<- array(NA, dim=c(length(exp_grp),length(adv_stv),length(years)),dimnames=list('exp_grp'=exp_grp,'stoves'=adv_stv,'years'=years))  ###############################################################################  ############### *Creates variables with current data* *and future projections* ################  ### expenditure per capita in ppp  v <- 'exp_ppp_pc'  exp_ppp_pc_RU <- adj_df(read.table(paste(filepath,'lam/data/upds/exp_ppp_pc_',scen,'.txt',sep=''), header=TRUE, sep='\t'))  grp_array[,v,] <- as.matrix(exp_ppp_pc_RU)  ### hh size  v <- 'hh_size'  hh_size_2005 <- read.table(paste(filepath,'lam/data/upds/R/Guatemala/2005/hh_size_2005.txt',sep=''), header=TRUE, sep='\t')  grp_array[,v,] <- hh_size_2005[,v]  reg_hh_size <- read.table(paste(filepath,'lam/data/upds/R/Guatemala/regress/regress_hh_size.txt',sep=''), header=TRUE, sep='\t')  y <- new_years  for (d in names(dyn_grp)) {  grp_array[d,v,y] <- reg_hh_size[match(dyn_grp[[d]],reg_hh_size[,'rururb']),'b']*log(grp_array[d,'exp_ppp_pc',y])+  reg_hh_size[match(dyn_grp[[d]],reg_hh_size[,'rururb']),'a']  }  ### if the new household size is bigger than 2, it's adjusted to 2  grp_array[,v,][grp_array[,v,]<2] <- 2  ### hh expenditure ###  grp_array[,'exp_ppp_hh',] <- grp_array[,'exp_ppp_pc',]*grp_array[,'hh_size',]  ### discount rate ###  v <- 'disc_rate'  disc_rate_2005 <- read.table(paste(filepath,'lam/data/upds/R/Guatemala/2005/disc_rate_2005.txt',sep=''), header=TRUE, sep='\t')  grp_array[,v,] <- disc_rate_2005[,v]  d <- names(dyn_grp)  grp_array[d,v,new_years] <- -0.162*log(grp_array[d,'exp_ppp_hh',new_years])+1.9558  ### cook ratio ###  v <- 'cook_ratio'  cook_ratio_2005 <- read.table(paste(filepath,'lam/data/upds/R/Guatemala/2005/cook_ratio_2005.txt',sep=''), header=TRUE, sep='\t')  grp_array[,v,] <- cook_ratio_2005[,v]  reg_cook_ratio <- read.table(paste(filepath,'lam/data/upds/R/Guatemala/regress/regress_cook_ratio.txt',sep=''), header=TRUE, sep='\t')  y <- new_years  for (d in names(dyn_grp)) {  grp_array[d,v,y] <- reg_cook_ratio[match(dyn_grp[[d]],reg_cook_ratio[,'rururb']),'a']*  (grp_array[d,'exp_ppp_pc',y])^reg_cook_ratio[match(dyn_grp[[d]],reg_cook_ratio[,'rururb']),'b'] }  ### cooking expenditure ###  v <- 'cook_exp'  grp_array[,v,] <- grp_array[,'cook_ratio',]*grp_array[,'exp_ppp_pc',]  for(d in names(dyn_grp)) {  max_year <- names(which.max(grp_array[d,v,]))  if (max_year!='2100')  { adj_year <- as.character(seq(as.numeric(max_year),2100,10))  grp_array[d,'cook_ratio',adj_year] <- grp_array[d,'cook_ratio',max_year]  grp_array[d,v,adj_year] <- grp_array[d,'cook_ratio',adj_year]*grp_array[d,'exp_ppp_pc',adj_year] }  }  #### Create Array of pre-credit adjustment discount rates ###  old_disc_rate<-grp_array[,'disc_rate',]  ######################################################  ### credit access case adjustment ###  if (ca!=0) { grp_array[,'disc_rate',policy_years][grp_array[,'disc_rate',policy_years]>(ca/100)] <- (ca/100) }  ### Targeted Stove Loan: Improved Biomass Stoves  disc_inbms <- array(NA, dim=c(length(exp_grp),length(years)),dimnames=list('exp_grp'=exp_grp,'years'=years))  disc_inbms <- grp_array[,'disc_rate',]  cainbms<-as.numeric(cainbms)  if (cainbms!=0) { disc_inbms[,policy_years][disc_inbms[,policy_years]>(cainbms/100)] <- (cainbms/100) }  ### Targeted Stove Loan: LPG Stoves  disc_gasst <- array(NA, dim=c(length(exp_grp),length(years)),dimnames=list('exp_grp'=exp_grp,'years'=years))  disc_gasst <- grp_array[,'disc_rate',]  cagasst<-as.numeric(cagasst)  #if (cagasst!=0) { disc_gasst[,policy_years][disc_gasst[,policy_years]>(cagasst/100)] <- (cagasst/100) }  if (cagasst!=0) {  cagasstpcts<-as.numeric(unlist(eval(strsplit(cagasstpcts,','))))  names(cagasstpcts)<-unlist(eval(strsplit(cagasstgrps,',')))  for (g in exp_grp){ disc_gasst[g,policy_years][disc_gasst[g,policy_years]>(cagasstpcts[g]/100)] <- (cagasstpcts[g]/100)}  }  ######################################################  ### cooking demand ###  v <- 'ck_dmd'  dmd_2005 <- read.table(paste(filepath,'lam/data/upds/R/Guatemala/2005/ck_dmd_2005.txt',sep=''), header=TRUE, sep='\t')  grp_array[,v,] <- dmd_2005[,v]  reg_dmd <- read.table(paste(filepath,'lam/data/upds/R/Guatemala/regress/regress_demand.txt',sep=''), header=TRUE, sep='\t')  y <- new_years  for (d in names(dyn_grp)) {  grp_array[d,v,y] <- (reg_dmd[match(dyn_grp[[d]],reg_dmd[,'rururb']),'b']*log(grp_array[d,'exp_ppp_pc',y])+  reg_dmd[match(dyn_grp[[d]],reg_dmd[,'rururb']),'a'])*grp_array[d,'hh_size',y]}  ### population data from text file ###  population <- adj_df(read.table(paste(filepath,'lam/data/upds/population.txt',sep=''), header=TRUE, sep='\t'))  grp_array[,'population',] <- as.matrix(population)  grp_array[,'hh_num',] <- grp_array[,'population',]/grp_array[,'hh_size',]  ### stove characteristics ###  stove_char <- adj_df(read.table(paste(filepath,'lam/data/upds/R/Guatemala/stv_char.txt',sep=''), header=TRUE, sep='\t'))  stove_char$fuel <- as.character(stove_char$fuel)  ### life year of stoves ###  lfyr_2005 <- stove_char$lfyr #replaced above line w this bc it means one less file to create (lfyr_2005.txt)  for (g in exp_grp) { stv_array[g,,'lfyr',] <- as.matrix(lfyr_2005) }  ### annualised stove price ###  for (s in stoves) {  stv_array[,s,'an_stv',] <- stove_char[s,'price']*grp_array[,'disc_rate',]*(1+grp_array[,'disc_rate',])^(stv_array[,s,'lfyr',])/  ((1+grp_array[,'disc_rate',])^stv_array[,s,'lfyr',]-1)}  # Biomass Stove Loan  if (cainbms!=0){  s<-'frwd_inbms'  stv_array[,s,'an_stv',] <- stove_char[s,'price']*disc_inbms*(1+disc_inbms)^(stv_array[,s,'lfyr',])/  ((1+disc_inbms)^stv_array[,s,'lfyr',]-1)}  # LPG Stove Loan  if (cagasst!=0){  s<-'lpgg_gasst'  stv_array[,s,'an_stv',] <- stove_char[s,'price']*disc_gasst*(1+disc_gasst)^(stv_array[,s,'lfyr',])/  ((1+disc_gasst)^stv_array[,s,'lfyr',]-1)}  ### fuel price ###  v <- 'fuel_price'  prices_2005 <- adj_df(read.table(paste(filepath,'lam/data/upds/R/Guatemala/2005/fuel_price_2005.txt',sep=''), header=TRUE, sep='\t'))  fuel_adj_2005 <- read.table(paste(filepath,'lam/data/upds/R/Guatemala/2005/fuel_adj_2005.txt',sep=''), header=TRUE, sep='\t')  options(warn=-1)  fuel_adj_2005[,'factor'] <- as.numeric(as.character(fuel_adj_2005[,'factor']))  options(warn= 1)  for (f in fuels) {  for (g in exp_grp) {  fuel_adj_array[g,f] <- fuel_adj_2005[fuel_adj_2005[,'fuel']==f & fuel_adj_2005[,'grp']==g,][['factor']]  }  }  for (s in stoves) {  stv_array[,s,v,'2005'] <- prices_2005[stove_char[s,'fuel'],]*fuel_adj_array[,stove_char[s,'fuel']]  for (y in new_years) {  stv_array[,s,v,y] <- MSG_prices[stove_char[s,'fuel'],y]*fuel_adj_array[,stove_char[s,'fuel']]  }  }  # create copy of stv_array with different name as basis for subsidy calculation  stv_price_temp <-stv_array  # lpg fuel subsidy  if (pslpgg!=0){  pslpggpcts<-as.numeric(unlist(eval(strsplit(pslpggpcts,','))))  names(pslpggpcts)<-unlist(eval(strsplit(pslpgggrps,',')))  for (g in unlist(eval(strsplit(pslpgggrps,',')))){  for (y in policy_years) {  stv_array[g,'lpgg_gasst', 'fuel_price', y]<-stv_price_temp[g,'lpgg_gasst','fuel_price',y]*(1-pslpggpcts[g]/100)  }  }  }  # elec fuel subsidy  if (pselec!=0){  pselecpcts<-as.numeric(unlist(eval(strsplit(pselecpcts,','))))  names(pselecpcts)<-unlist(eval(strsplit(pselecgrps,',')))  for (g in unlist(eval(strsplit(pselecgrps,',')))){  for (y in policy_years) {  stv_array[g,'elec_elest', 'fuel_price', y]<-stv_price_temp[g,'elec_elest','fuel_price',y]*(1-pselecpcts[g]/100)  }  }  }  ### total cooking price ###  v <- 'cook_price'  for (s in stoves) {  stv_array[,s,v,] <- (stv_array[,s,'fuel_price',]/stove_char[s,][['eff']]) +  (stv_array[,s,'an_stv',]/grp_array[,'ck_dmd',])  }  ###############################################################  ###################### *Estimating fuel/stove demands by groups* ######################  ### create a matrix with coefficients ###  coeff_exp_grp <- read.table(paste(filepath,'lam/data/upds/R/Guatemala/2005/coeff_exp_grp_2005.txt',sep=''), header=TRUE, sep='\t')  for (s in adv_stv) {  for (g in exp_grp) {  stv_coeff_array[g,s,'a',] <- coeff_exp_grp[coeff_exp_grp[,'stove']==s & coeff_exp_grp[,'grp']==g,][['a']]  stv_coeff_array[g,s,'b',] <- coeff_exp_grp[coeff_exp_grp[,'stove']==s & coeff_exp_grp[,'grp']==g,][['b']]  }  }  ### adjust coefficients 'a' for the future years for dynamic groups to reflect increase in their purchasing power  for (tech in adv_stv) {  for (g in names(dyn_grp)) {  ### increase in purchasing power compared to 2005 is added to the coefficient a  stv_coeff_array[g,tech,'a',] <- stv_coeff_array[g,tech,'a',]+(grp_array[g,'cook_exp',]-grp_array[g,'cook_exp','2005'])  }  }  for (s in adv_stv){  constraint_array[,s,]<-grp_array[,'ck_dmd',]  }  s<-'frwd_inbms'  old_yr<-c('2005','2010')  constraint_array[,s,old_yr]<-grp_array[,'ck_dmd',old_yr]*0.1  ########################################  ### calculate demands for non-three stoves using demand curves ###  v <- 'orig_dmd'  for (s in adv_stv) {  demand_array[,s,v,] <- (stv_array[,s,'cook_price',]/stv_coeff_array[,s,'a',])^(1/stv_coeff_array[,s,'b',])  ## demand adjustment for the dynamic groups  demand_array[,s,v,] <- demand_array[,s,v,]*(grp_array[,'ck_dmd',]/grp_array[,'ck_dmd','2005'])  ## if demands are NA, its replaced by 0  demand_array[,s,v,][is.na(demand_array[,s,v,])] <- 0  }  # Tier 1 Demand  ## Tier 1 cooking price array  tier1_cookprice <- array(NA, dim=c(length(exp_grp),length(tier1),length(years)),  dimnames=list('exp_grp'=exp_grp,'stoves'=tier1,'years'=years))  for (s in tier1){  for (g in exp_grp){  for (y in years){  tier1_cookprice[g,s,y] <- stv_array[g,s,'cook_price',y]  }  }  }  ### Calculate Tier 1 Demand  for (g in exp_grp){  for (y in years){  totdmd<-0  # set s = cheapest fuel for that tier, exp_grp, and year  s <- names(tier1_cookprice[g,,y])[order(tier1_cookprice[g,,y])[1]]  ########################################### 5% BUFFER ##########################  #tier1_cookprice[g,s,y]>0 &&  if (y!='2005'){  if ((abs(tier1_cookprice[g,s,y]-tier1_cookprice[g,s_old,y])/tier1_cookprice[g,s,y])<0.05){  s<-s_old  }  }  ##################################################################################  demand_array[g,s,'demand_UE',y]<-min(demand_array[g,s,'orig_dmd',y],constraint_array[g,s,y],grp_array[g,'ck_dmd',y])  totdmd<-demand_array[g,s,'demand_UE',y]  s_old <- s  # loop through stove types in that tier from 2nd cheapest to most expensive  tcp <- tier1_cookprice[g,,y][names(tier1_cookprice[g,,y])!=s]  for (i in order(tcp)) {  s2<-names(tcp)[i]  if (min(constraint_array[g,s2,y],demand_array[g,s2,'orig_dmd',y])<=totdmd){  demand_array[g,s2,'demand_UE',y]<-0  } else if (demand_array[g,s2,'orig_dmd',y]<min(constraint_array[g,s2,y],grp_array[g,'ck_dmd',y])){  demand_array[g,s2,'demand_UE',y]<-demand_array[g,s2,'orig_dmd',y]-totdmd  } else {demand_array[g,s2,'demand_UE',y]<-min(constraint_array[g,s2,y],grp_array[g,'ck_dmd',y])-totdmd}  totdmd<-totdmd+demand_array[g,s2,'demand_UE',y]  }  }  }  # Total Tier 1 demand  tier1_totdmd<-array(NA, dim=c(length(exp_grp),length(years)),  dimnames=list('exp_grp'=exp_grp,'years'=years))  for (y in years){  for (g in exp_grp){  tier1_totdmd[g,y]<-sum(demand_array[g,,"demand_UE",y],na.rm=T)  }  }  ### Tier 2 cooking price array  tier2_cookprice <- array(NA, dim=c(length(exp_grp),length(tier2),length(years)),  dimnames=list('exp_grp'=exp_grp,'stoves'=tier2,'years'=years))  for (s in tier2){  for (g in exp_grp){  for (y in years){  tier2_cookprice[g,s,y] <- stv_array[g,s,'cook_price',y]  }  }  }  ### Calculate Tier 2 Demand  for (y in years){  for (g in exp_grp){  s<-'frwd_inbms'  if ((demand_array[g,s,'orig_dmd',y])>min(constraint_array[g,s,y],(round(grp_array[g,'ck_dmd',y],4)-round(tier1_totdmd[g,y],4))))  {demand_array[g,s,'demand_UE',y]<-min(constraint_array[g,s,y],(round(grp_array[g,'ck_dmd',y],4)-round(tier1_totdmd[g,y],4)))}  else {demand_array[g,s,'demand_UE',y]<-demand_array[g,s,'orig_dmd',y]}  }  }  # Total advanced stove demand  tier12_totdmd<-array(NA, dim=c(length(exp_grp),length(years)),  dimnames=list('exp_grp'=exp_grp,'years'=years))  for (y in years){  for (g in exp_grp){  tier12_totdmd[g,y]<-sum(demand_array[g,,"demand_UE",y],na.rm=T)  }}  ### Three stone stove demand ###  f='frwd_three'  d='demand_UE'  for (y in years){  for (g in exp_grp){  demand_array[g,f,d,y]<-round(grp_array[g,'ck_dmd',y],4)-round(tier12_totdmd[g,y],4)  }  }  ### adjust if estimate exceeds expected demand  MESSAGE_demand[,,,'GJ'] <- demand_array[,,'demand_UE',]  for (y in years) {  ## set all the negative values to zero  MESSAGE_demand[,,y,'GJ'][MESSAGE_demand[,,y,'GJ']<0] <- 0  }  ### convert to GWyr and multiply by number of hhs ###  for (s in stoves) { MESSAGE_demand[,s,,'GWyr']<- MESSAGE_demand[,s,,'GJ']*grp_array[,'hh_num',]*1e6 }  MESSAGE_demand[,,,'GWyr'] <- MESSAGE_demand[,,,'GWyr']*(1/3.6)*(1/1000)*(1/8760)  for (grp in exp_grp) { MESSAGE_demand[grp,,,'GWyr_FE'] <- MESSAGE_demand[grp,,,'GWyr']/stv_eff }  for (s in all_stv){  MESSAGE_demand[,s,,'GJ/cap']<-MESSAGE_demand[,s,,'GJ']/grp_array[,'hh_size',]  }  capture.output(print(round(MESSAGE_demand,4), print.gap=3),file=paste(filepath2,coreid,'_MESSAGE_demand.upd',sep =''))  # This is the effective demand output by expenditure group, and period: 'MESSAGE_demand'  # This data can be used to estimate emissions and policy costs  }  #-----------------  # END  #-----------------  -----------------  INPUT DATA FILES  -----------------  fuel_price_2005.txt  fuel fuel_price  frwd 13.57719  lpgg 55.41017  elec 122.8793  exp_ppp_pc_geam.txt  grp 2005 2010 2020 2030 2040 2050 2060 2070 2080 2090 2100  R1 429.7 429.7 429.7 429.7 429.7 429.7 429.7 429.7 429.7 429.7 429.7  R2 1200.7 1200.7 1200.7 1200.7 1200.7 1200.7 1200.7 1200.7 1200.7 1200.7 1200.7  R3 4453.0 4652.7 6453.4 9940.4 15062.6 21790.1 29993.2 40104.7 52562.1 68264.7 88388.2  U1 1115.8 1115.8 1115.8 1115.8 1115.8 1115.8 1115.8 1115.8 1115.8 1115.8 1115.8  U2 7263.5 7282.5 10548.1 15573.3 21301.4 27256.3 33033.3 38969.1 45215.1 52149.5 60106.2  hh_size_2005.txt  grp hh_size  R1 6.4212  R2 5.6894  R3 4.8334  U1 5.3383  U2 4.2372  regress_hh_size.txt  rururb a b  exp_grpR20 11.7622 -0.8465  exp_grpU20 9.8379 -0.6487  disc_rate_2005.txt  grp disc_rate  R1 0.6723  R2 0.5501  R3 0.3951  U1 0.5791  U2 0.3514  cook_ratio_2005.txt  fuel cook_ratio  R1 0.2218  R2 0.1037  R3 0.0371  U1 0.1269  U2 0.0258  regress_cook_ratio.txt  rururb a b  exp_grpR20 22.07 -0.755  exp_grpU20 38.165 -0.811  ck_dmd_2005.txt  grp ck_dmd  R1 13.7751  R2 10.8580  R3 7.9409  U1 13.7751  U2 8.7261  regress_demand.txt  rururb a b  exp_grpR20 1.6812 0.0809  exp_grpU20 2.5136 -0.1083  population.txt  grp 2005 2010 2020 2030 2040 2050 2060 2070 2080 2090 2100  R1 1.716 1.781 0.991 0.409 0.176 0.073 0.026 0.001 0.000 0.000 0.000  R2 2.289 2.428 2.041 1.334 0.859 0.574 0.411 0.299 0.207 0.143 0.097  R3 2.708 3.056 5.101 6.799 7.604 7.864 8.004 7.986 7.854 7.647 7.386  U1 1.223 1.447 1.168 0.788 0.539 0.376 0.273 0.190 0.118 0.056 0.003  U2 4.774 5.664 8.790 12.362 15.707 18.593 21.366 23.568 25.101 25.988 26.287  stv_char.txt  stove fuel eff lfyr price  frwd_three frwd 0.15 3 0.00  frwd_inbms frwd 0.25 10 100.00  lpgg_gasst lpgg 0.6 15 164.96  elec_elest elec 0.75 15 205.56  coeff_exp_grp_2005.txt  stove grp a b  frwd_inbms R1 111.1851 -0.5985  frwd_inbms R2 133.6494 -0.6271  frwd_inbms R3 91.8983 -0.4151  frwd_inbms U1 100.9593 -0.4258  frwd_inbms U2 61.0530 -0.2754  lpgg_gasst R1 53.0895 -0.4133  lpgg_gasst R2 98.6868 -0.3251  lpgg_gasst R3 110.0172 -0.2363  lpgg_gasst U1 139.2596 -0.3207  lpgg_gasst U2 136.8973 -0.2113  elec_elest R1 53.0895 -0.4133  elec_elest R2 98.6868 -0.3251  elec_elest R3 110.0172 -0.2363  elec_elest U1 139.2596 -0.3207  elec_elest U2 136.8973 -0.2113 |
| --- |
