# Supplementary material for: Outlook for modern cooking energy access in Central America
Source: PLoS One. 2018 Jun 8;13(6):e0197974. doi: 10.1371/journal.pone.0197974 (PMC5993280; doi:10.1371/journal.pone.0197974)
Supplement: S6 Table — (DOCX) [file pone.0197974.s006.docx]

Table S6: Additional scenario results on share of population using different stoves

| **Share of population with access in Guatemala** | | | | | | | | |
| --- | --- | --- | --- | --- | --- | --- | --- | --- |
| **Scenario/ Year** | **All/ 2010** | **NNP/ 2030** | **G_ICS_100/ 2030** | **TG_LPG_50/ 2030** | **TG_LPG_100/ 2030** | **G_EIS_100/ 2030** | **TT_LPG_20/ 2030** | **TT_LPG_50/ 2030** |
| **3-Stone** | 50% | 8% | 7% | 8% | 8% | 8% | 7% | 3% |
| **ICS** | 10% | 13% | 13% | 15% | 12% | 13% | 4% | 3% |
| **LPG** | 40% | 79% | 79% | 77% | 80% | 79% | 89% | 95% |
| **EIS** | 0% | 0% | 0% | 0% | 0% | 0% | 0% | 0% |
| **Share of population with access in Honduras** | | | | | | | | |
| **Scenario/ Year** | **All/ 2010** | **NNP/ 2030** | **G_ICS_100/ 2030** | **TG_LPG_50/ 2030** | **TG_LPG_100/ 2030** | **G_EIS_100/ 2030** | **TT_LPG_20/ 2030** | **TT_LPG_50/ 2030** |
| **3-Stone** | 55% | 14% | 12% | 13% | 12% | 13% | 7% | 1% |
| **ICS** | 10% | 13% | 15% | 9% | 7% | 13% | 7% | 0% |
| **LPG** | 35% | 73% | 73% | 79% | 82% | 29% | 86% | 98% |
| **EIS** | 0% | 0% | 0% | 0% | 0% | 45% | 0% | 0% |
| **Share of population with access in Nicaragua** | | | | | | | | |
| **Scenario/ Year** | **All/ 2010** | **NNP/ 2030** | **G_ICS_100/ 2030** | **TG_LPG_50/ 2030** | **TG_LPG_100/ 2030** | **G_EIS_100/ 2030** | **TT_LPG_20/ 2030** | **TT_LPG_50/ 2030** |
| **3-Stone** | 58% | 10% | 1% | 10% | 10% | 10% | 9% | 6% |
| **ICS** | 2% | 29% | 38% | 27% | 25% | 29% | 11% | 0% |
| **LPG** | 40% | 61% | 61% | 63% | 66% | 61% | 80% | 94% |
| **EIS** | 0% | 0% | 0% | 0% | 0% | 0% | 0% | 0% |
