# Supplementary material for: Outlook for modern cooking energy access in Central America
Source: PLoS One. 2018 Jun 8;13(6):e0197974. doi: 10.1371/journal.pone.0197974 (PMC5993280; doi:10.1371/journal.pone.0197974)
Supplement: S1 Fig — (PDF) [file pone.0197974.s007.pdf]

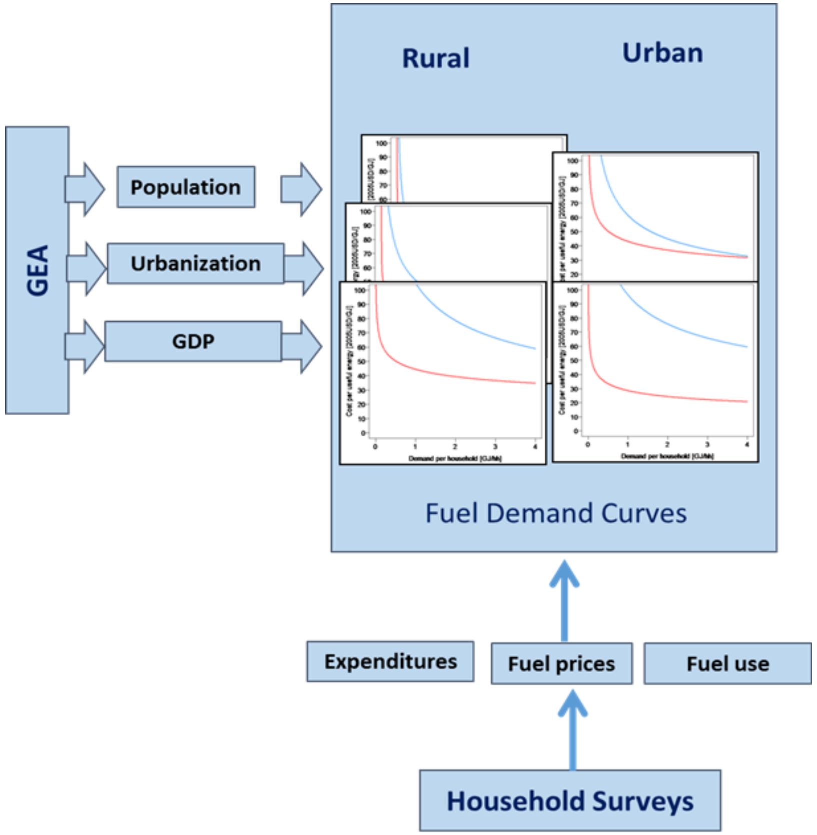

Fig S1: Schematic overview of model and data sources (Note: A public version of the model is available at http://data.ene.iiasa.ac.at/MESSAGE-Access/).
