# Supplementary figures and images for: Outlook for modern cooking energy access in Central America
Source: PLoS One. 2018 Jun 8;13(6):e0197974. doi: 10.1371/journal.pone.0197974 (PMC5993280; doi:10.1371/journal.pone.0197974)

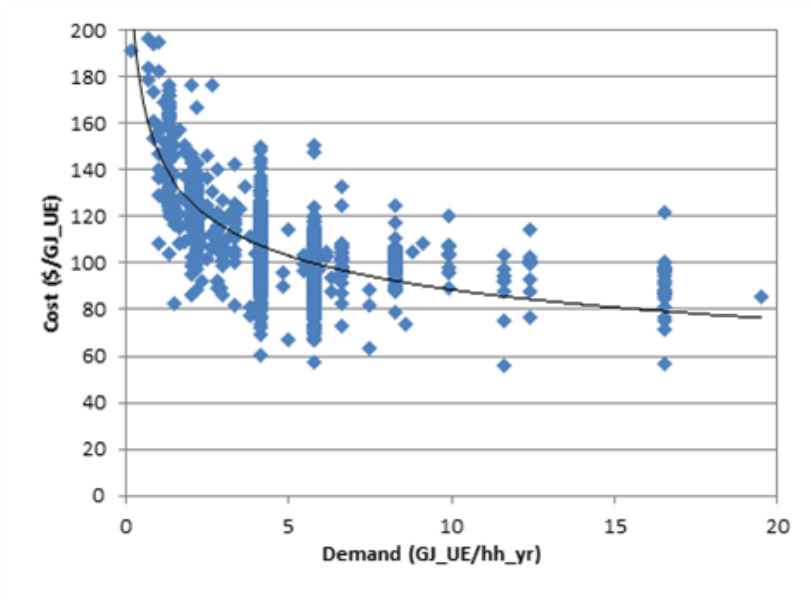

*Fig S2: Example demand curve for LPG in Guatemala for the U2 expenditure group*

Supplement: S2 Fig — (PDF) [file pone.0197974.s008.pdf]
